# Development of a database of capsaicinoid contents in foods commonly consumed in Korea

**DOI:** 10.1002/fsn3.1785

**Published:** 2020-07-16

**Authors:** Hoyoun Cho, Youngjoo Kwon

**Affiliations:** ^1^ Department of Food Science and Engineering Ewha Womans University Seoul Korea

**Keywords:** capsaicinoid, chili pepper, consumption, database, health effects, red pepper powder

## Abstract

Chili peppers (*Capsicum annuum* L.) are widely consumed worldwide, and the health benefits of capsaicinoids (the active compounds in chili peppers) have been suggested. However, the link between capsaicinoid consumption and the risk of certain cancers remains controversial. Capsaicinoid consumption level is an important determinant of its potential health effects. This study sought to construct a database of capsaicinoid contents in foods commonly consumed in Korea (CAPKO) to enable a more reliable estimation of capsaicinoid intake. Capsaicinoid‐containing foods were identified from the Korea National Health and Nutrition Examination Survey datasets and divided into eight categories: chili peppers, red pepper powder, hot sauce, kimchi, salted seafood, red pepper paste, instant noodles, and convenience foods other than instant noodles. The capsaicinoid contents of primary capsaicinoid sources (chili peppers, red pepper powder, and hot sauce) were estimated from the literature. For the remaining food categories, the contents of primary capsaicinoid sources were identified from standardized recipes (kimchi) or food labels (salted seafood, red pepper paste, and convenience foods other than instant noodles). Then, capsaicinoid contents were estimated by calculation using the identified capsaicinoid source contents and the estimated capsaicinoid content in these sources. This information was unavailable for instant noodles, and capsaicinoid content was measured by HPLC analyses. This study developed the CAPKO database, which includes a variety of foods with varying levels of spiciness, which can be used in combination with dietary surveys to estimate capsaicinoid intakes. Therefore, this study established a framework for future database development for other compounds with potential health effects.

## Graphical abstract

This study developed a database of capsaicinoid content in foods commonly consumed in Korea to enable a more reliable estimation of capsaicinoid intake. Food items that contained primary capsaicinoid sources (chili peppers, red pepper powder, and hot sauce) were selected using the Korea National Health and Nutrition Examination Survey datasets and divided into eight categories: chili peppers, red pepper powder, hot sauce, kimchi, salted seafood, red pepper paste, instant noodles, and convenience foods other than instant noodles. Capsaicinoid contents in primary capsaicinoid sources were estimated from the literature. For the remaining food categories, the contents of primary capsaicinoid sources were identified from standard recipes or food labels (e.g., kimchi, salted seafood, red pepper paste, and convenience foods other than instant noodles). Then, capsaicinoid contents were estimated by calculation using the identified primary capsaicinoid source contents and the estimated capsaicinoid contents in primary capsaicinoid sources. When the content of the primary capsaicinoid source was unidentifiable (e.g., instant noodles), capsaicinoid levels were analyzed via HPLC analyses.

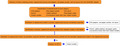

## 1 INTRODUCTION

Chili peppers (*Capsicum annuum* L.) are among the world's most widely used spices and are particularly popular in Asia, Latin America, and Africa. Moreover, spicy foods are the mainstay of many cuisines across the globe. *C. annuum* varieties encompass a wide range of chili pepper shapes and sizes, both mild and hot (Wang, Xia, Wang, Luo, & Huang, 2009).

The pungency or spiciness of chili peppers is directly correlated with the concentration of capsaicinoids present in the fruit. The most commonly occurring capsaicinoids are capsaicin (8‐methyl‐*N*‐vanillyl‐6‐nonenamide) and dihydrocapsaicin (8‐methyl‐*N*‐vanillylnonanamide), which together constitute approximately 90% of all capsaicinoids (Barbero, Liazid, Azaroual, Palma, & Barroso, 2016). Capsaicin is an irritant that produces both thermal and burning sensations upon contact with oral or skin mucosa (Caterina et al., 1997). Capsaicin‐induced irritation is mediated by transient receptor potential vanilloid I (TRPV1) or vanilloid receptor 1 (Julius, 2013). TRPV1 is a nonselective cation channel that is also activated by heat (>42°C), protons, and physical abrasion (Caterina et al., 1997). The activation of TRPV1 allows the preferential influx of Ca^2+^ cation, resulting in the depolarization of neurons and the propagation of nociceptive signals to the brain and spinal cord (Caterina et al., 1997; Cui et al., 2006).

The topical application of capsaicin has been extensively studied as an effective pain management alternative (Fattori, Hohmann, Rossaneis, Pinho‐Ribeiro, & Verri, 2016). When TRPV1‐expressing sensory nerve fibers are exposed to high or repeated capsaicin doses, the TRPV1 receptors undergo a refractory state (desensitization) that inhibits the receptor function (Comunanza, Carbone, Marcantoni, Sher, & Ursu, 2011). Capsaicin‐induced desensitization causes the sensory neurons to become less responsive to endogenous TRPV1 agonists, resulting in analgesia (Anand & Bley, 2011).

TRPV1 occurs not only in nociceptive sensory neurons but also in vascular endothelial cells, hepatocytes, adipocytes, smooth muscle cells, fibroblasts, T cells, mast cells, and astrocytes (Gunthorpe & Szallasi, 2008). This widespread expression of TRPV1 in vivo suggests its potential role in these cells as a modulator of intracellular calcium levels (McCarty, DiNicolantonio, & O'Keefe, 2015). In fact, capsaicin has been linked to many health benefits including protection from cardiovascular disease, insulin sensitivity improvement, and weight gain amelioration mainly through TRPV1 activation (Baskaran et al., 2017; Ma et al., 2011; Marshall et al., 2013; McCarty et al., 2015). Additionally, anticancer activity of capsaicin has been shown in various types of cancer, including lung, prostate, and breast cancer, partly due to its ability to induce cell‐cycle arrest or apoptosis (Clark & Lee, 2016).

Nonetheless, capsaicin has also been suggested to possess co‐carcinogenic effects in skin cancer (Bode & Dong, 2011). Exposure to capsaicin promoted the growth of skin tumors initiated by 7,12‐dimethyl benz(a)anthracene or 12‐O‐tetradecanoylphorbol‐13‐acetate in mice, although capsaicin alone did not develop skin cancer (Liu et al., 2015). Moreover, some epidemiological studies have suggested an association between high chili pepper consumption and an increased cancer risk, especially gastric cancer (Chen et al., 2017; Lopez‐Carrillo, Hernandez Avila, & Dubrow, 1994). However, the evidence for increased risk of cancer by capsaicinoid consumption is insufficient. No clear genotoxicity has been associated with capsaicinoid exposure, and potential confounding factors (e.g., food contaminants) in previous epidemiological studies have been pointed out (Bley, Boorman, Mohammad, McKenzie, & Babbar, 2012). A meta‐analysis study reported that moderate capsaicin consumption was associated with protection from gastric cancer development, whereas medium or high consumption increased the risk of this disease (Pabalan, Jarjanazi, & Ozcelik, 2014). Therefore, a clear link between capsaicin or capsaicinoids and cancer risk remains to be established.

Given that the amount of capsaicinoid consumption might determine its potential health effects, a means to accurately estimate capsaicinoid consumption would be a useful risk assessment tool. Previous capsaicinoid consumption estimation has mainly relied on characterizing the frequency of chili pepper or chili pepper‐containing food consumption without accounting for capsaicinoid content (Chen et al., 2017; Lopez‐Carrillo et al., 1994). The lack of a comprehensive database of the capsaicinoid contents of different food items makes it difficult to determine the association between capsaicinoid consumption and health effects.

The demand for spicy foods in Korea has accelerated the development of various spicy products, such as hot‐flavored instant noodles. Various cultivars of “gochu” (chili pepper) are available in Korea, all of which vary in spiciness intensity (Kim, Park, & Hwang, 2002). Red pepper powder (“gochugaru”) is a Korean chili‐based product that is sold as powder or flakes and is commonly used in Korean cuisine. This product is a major condiment in spicy food and a primary source of capsaicinoids in the Korean diet. Its spiciness largely depends on its pepper cultivar composition (Lee et al., 2011) and cultivation location (Lee et al., 2013). Capsaicinoid consumption may be reasonably high for certain individuals who consume spicier versions of the same foods by adding more ingredients that contain capsaicinoids (e.g., red pepper powder) or using ingredients that contain high levels of capsaicinoids (e.g., red pepper powder with higher capsaicinoid content). These variations highlight the importance of developing a database of capsaicinoid content in commonly consumed food items in Korea to allow for a reliable estimation of capsaicinoid intakes and the identification of individuals with high capsaicinoid consumption.

Therefore, the CAPKO database was created in this study to estimate the capsaicinoid content of foods commonly consumed in Korea. The information provided by this database will allow for the evaluation of capsaicinoid intakes in Korea and the identification of subpopulations that consume high amounts of capsaicinoids to determine associated health effects. Moreover, this study establishes a methodological framework for the development of databases for other compounds with potential health effects.

## 2 MATERIALS AND METHODS

### 2.1 Selection of capsaicinoid‐containing food items

Capsaicinoids in the Korean diet are primarily derived from chili peppers, red pepper powder, and hot sauce. Therefore, food items that contained these components were identified using the Korea National Health and Nutrition Examination Survey (KNHANES) datasets. The KNHANES is a nationwide survey program conducted by the Korea Centers for Disease Control and Prevention to assess the health and nutritional status of the Korean population. This representative, nationwide, and cross‐sectional survey annually gathers information from approximately 10,000 individuals aged ≥1 year (Kweon et al., 2014). The nutritional component of the KNHANES assesses dietary behaviors, food frequency, and food intake (Kweon et al., 2014), and therefore serves as a useful resource to estimate dietary exposure (Kwon, 2014).

Twenty‐four‐hour dietary recall data from the KNHANES survey from 2012 to 2016 were examined in terms of the “coded food name” to identify common food items in the Korean diet that contain chili peppers, red pepper powder, and hot sauce. The 178 resulting capsaicinoid‐containing foods identified in the Korean diet were then classified into eight food categories: chili peppers, red pepper powder, hot sauce, kimchi, salted seafood, red pepper paste, instant noodles, and convenience foods other than instant noodles.

### 2.2 Estimation of capsaicinoid content in selected foods

**FIGURE 1:**
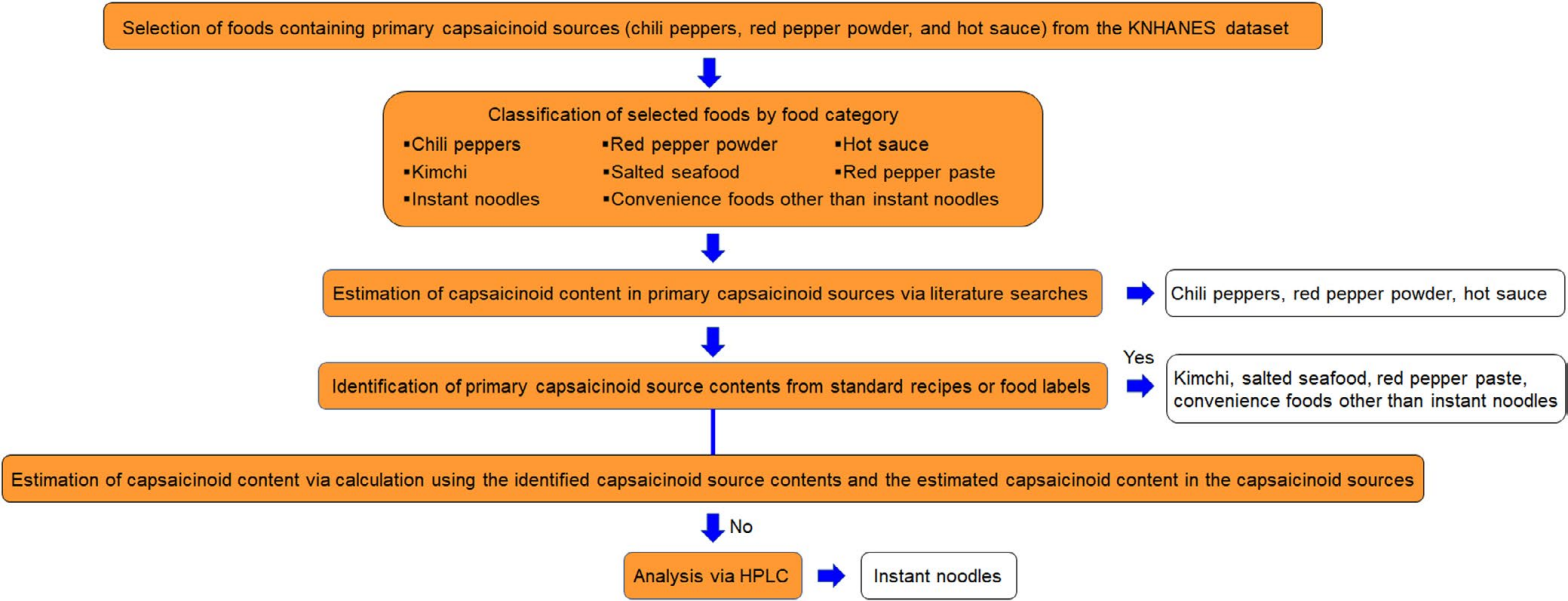
Flow chart of the steps taken to develop the database of capsaicinoid contents in foods commonly consumed in Korea

Figure 1 illustrates a flow chart of the steps taken to develop the CAPKO database. To estimate the capsaicinoid content of selected food items, the capsaicinoid levels of primary capsaicinoid sources (chili peppers, red pepper powder, and hot sauce) were first compiled from the literature. For the remaining food categories, their major capsaicinoid sources were determined, and capsaicinoid contents were identified from standard recipes or food labels. Afterward, the capsaicinoid contents of kimchi, salted seafood, red pepper paste, and convenience foods other than instant noodles were estimated via calculation. Capsaicinoid levels in kimchi and red pepper paste were also available from the literature, and these values were compared with the estimated values to validate calculations in this study. When the content of the primary capsaicinoid source was unidentifiable (e.g., instant noodles), capsaicinoid levels were analyzed via high‐performance liquid chromatography (HPLC) analyses.

#### 2.2.1 Estimation of capsaicinoid content through literature searches

The capsaicinoid contents of chili peppers, red pepper powder, and hot sauce were estimated by literature searches. Chili peppers are often consumed fresh, and four types are mainly produced for fresh consumption. Red pepper powder is obtained by processing red chili peppers. However, the chili pepper cultivars for red pepper powder are different from those produced for fresh consumption. Hot sauces can be made from various kinds of peppers such as Tabasco pepper (*Capsicum frutescens*), cayenne pepper (*Capsicum annuum*), and habanero pepper (*Capsicum chinense*), as well as mixtures of these varieties. Therefore, the capsaicinoid contents of chili peppers, red pepper powder, and hot sauce can vary considerably.

Capsaicinoid contents in chili peppers, red pepper powder, and hot sauce were obtained from previous reports that analyzed capsaicinoids using reliable analytical methods such as HPLC and gas chromatography (GC). Capsaicinoid content (combined capsaicin and dihydrocapsaicin levels) was expressed as milligrams of capsaicinoid in 100 g of wet weight or dry weight, depending on the way they are consumed; wet weight was used for Korean chili pepper as well as hot sauce, whereas dry weight was used for red pepper powder.

When capsaicin or dihydrocapsaicin content was only available on a dry weight basis in chili peppers, the values were converted to a wet weight basis after correcting for water contents in fresh and dried peppers using data available from the Korean Food Composition Table (KFCT, version nine) (RDA, 2017). The KFCT is a database of energy and nutrient contents in foods commonly consumed in Korea. The moisture correction factor was calculated using the following formula: (100 – fresh pepper water content)/ (100 – dried pepper water content). When dihydrocapsaicin levels were not measured, the ratio of capsaicin to dihydrocapsaicin for a given cultivar (Korean chili pepper or Chengyang chili pepper) derived from other studies that analyzed both capsaicinoids was used to estimate dihydrocapsaicin level.

Red pepper powder is a major source of capsaicinoids in many foods, and therefore contributes significantly to capsaicinoid intake. Additionally, capsaicinoid content in red pepper powder can be affected by many factors including region, cultivar, and drying conditions (Kim et al., 2002; Lee et al., 2013; Wang et al., 2009), resulting in variation in red pepper powder spiciness (Yu, Choi, & Lee, 2009). In this study, literature covering the capsaicinoid content of red pepper powders produced in Korea was comprehensively searched. A total of six studies used GC or HPLC to analyze the capsaicinoid levels of various red pepper powders consumed in Korea. One study provided only a mean capsaicinoid content (35.3 mg/100 g of red pepper powder) for a variety of red pepper powders that were purchased from different locations in Korea (Dang et al., 2018). Individual values from five other studies were used for estimations in this study. Capsaicinoid levels in red pepper powder were estimated in terms of five levels of spiciness. These red pepper powder capsaicinoid concentrations were used throughout this study to estimate the capsaicinoid content in kimchi and other foods that contain red pepper powder as their primary capsaicinoid source.

#### 2.2.2 Estimation of capsaicinoid content via calculation

Red pepper powder is a major capsaicinoid source in kimchi, salted seafood, red pepper paste, and many other processed foods. In addition to red pepper powder, convenience foods other than instant noodles contain hot sauce as a major source of capsaicinoids. For each food category, this study examined whether the amount of these capsaicinoid sources can be identified using standard recipes or food labels that provided information on their major ingredients and their content, as mandated by Korean law.

For example, standardized recipes for different types of kimchi were searched. The weights of the main ingredients were combined, and red pepper powder portion (%) was calculated. When standardized recipes only provided the fresh weight (rather than drained weight) of cabbage or radishes, they were corrected for weight loss (approximately 10%) due to moisture loss during the salting and draining processes (Han & Seok, 1998). Capsaicinoid content was further corrected to reflect the degradation by microorganisms during fermentation (Ku, Park, & Park, 2004). For kimchi types for which standardized recipes were unavailable, their red pepper powder content was estimated from the red pepper powder content of other kimchi made from vegetables with similar mechanical properties.

For the estimation of capsaicinoid source contents (%) in salted seafood, red pepper paste, and convenience foods other than instant noodles (e.g., kimchi dumpling and spicy canned tuna), products available in the market were examined, and the food labels were reviewed to identify red pepper powder and hot sauce contents. Red pepper powder contents were averaged from different products within the same food item classification.

Afterword, capsaicinoid content was calculated using the identified capsaicinoid sources contents (red pepper powder and/or hot sauce) based on standard recipes and food labels coupled with the capsaicinoid content reported in the literature. The estimated capsaicinoid levels in capsaicinoid sources were multiplied by the capsaicinoid source contents and divided by 100. When foods contained more than one capsaicinoid source (e.g., spicy canned tuna), the capsaicinoid levels derived from each capsaicinoid source were combined to estimate capsaicinoid contents. Capsaicinoid content was expressed as milligrams of capsaicinoids per 100 g of consumable food.

#### 2.2.3 Measurement of capsaicinoid content via HPLC analysis

Capsaicinoids in instant noodles are contained exclusively in the seasoning powders or liquids provided with each noodle block. Major sources of capsaicinoids in seasonings include red pepper powder and chili oleoresin. Hot flavors can originate from different combinations of chili peppers; however, this information is often proprietary. Therefore, the capsaicinoid level in instant noodles was determined via HPLC analysis of the seasonings provided with the noodle block. Capsaicinoid levels were expressed as milligrams of capsaicinoids per 100 g of seasoning. Seasoning size (weight) varied among the examined products, and therefore, the capsaicinoid content was also expressed per seasoning packet (one serving).

A total of 24 instant noodle products were selected upon considering their spiciness, market share, and inclusion in the KNHANES. The samples included 16 regular (packaged in a bag) and 8 cup noodles produced by four major manufacturers (Nongshim, NS; Ottogi, OTG; Paldo, PD; and Samyang, SY). All items were purchased from local markets in Seoul, Korea. Analytical standards of capsaicin (≥98.5%) and dihydrocapsaicin (≥97.0%) were purchased from Sigma‐Aldrich. Stock solutions of capsaicin and dihydrocapsaicin prepared in methanol were stored in the dark at −20°C and further diluted to the desired concentration with 75% methanol to provide a daily working standard. All other reagents and solvents used were of analytical or HPLC grade.

Capsaicinoids were extracted from seasoning powders or liquids via the heating‐block method, as described in a previous study with minor modifications (Namgung, Lee, & Ha, 2013). Briefly, the samples (powder, 3 g; liquid, 1 g) were heated in 75% (powder) or 100% (liquid) methanol at 95°C for 1 hr. The extraction was performed three times for each sample. The extracts were collected and centrifuged at 6,200 *g* for 10 min to remove particulates. Supernatants were adjusted to 25 ml with methanol and filtrated into an HPLC sample vial through a 0.2‐μm syringe filter.

HPLC analyses were carried out on a Gilson HPLC system equipped with an auto‐injector and a UV/VIS detector set at 280 nm (Gilson, Inc.). The capsaicinoids were separated with a Zorbax 300SB‐C18 (4.6 × 250 mm, 5 μm) column (Agilent Technologies, Inc.) by elution with isocratic acetonitrile/water containing 0.1% acetic acid 40/60 (v/v) with a 1 ml/min flow rate. The injection volume was 20 μl.

The capsaicin standard was dissolved in 75% methanol at concentrations of 0.313, 1.25, 5, 20, and 80 μg/ml. Dihydrocapsaicin was dissolved in 75% methanol at concentrations of 0.625, 2.5, 10, and 40 μg/ml. The standard solutions were analyzed under the same analytical conditions as the samples, and the obtained standard curve plots (peak area against concentration) were used to quantify the capsaicin or dihydrocapsaicin level in instant noodle seasonings.

For the recovery test, 24 μg of capsaicin and 12 μg of dihydrocapsaicin were spiked into the seasonings of soy flour (flavored) noodles (no capsaicinoid), NS NGR (Neoguri; powder) and PD BBM (Bibim myun; liquid), and their recovery rates were 88.7 ± 8.65, 96.8 ± 12.64, and 86.1 ± 7.37%, for capsaicin and 100.9 ± 14.92, 99.6 ± 3.34, and 86.1 ± 7.37% for dihydrocapsaicin, respectively.

## 3 RESULTS AND DISCUSSION

### 3.1 Chili peppers

Four main types of chili peppers (“gochu”) are cultivated for fresh consumption in Korea (Park et al., 2019). The most common cultivar is Korean chili pepper (“Nokguang gochu”) or Korean hot pepper, also known as Korean dark green pepper or Korean red pepper depending on its color/ripening stages (the peppers are dark green when unripe). Cheongyang chili pepper (“Cheongyang gochu”) is the hottest cultivar (Hwang et al., 2011). Shishito pepper (“Kkawri gochu”) is a mildly hot East Asian variety that is also known as ground cherry pepper in Korea due to its wrinkled surface (Park et al., 2019). Cucumber chili peppers (“Oi gochu”) have a crunchy texture and are not spicy (Park et al., 2019).

Capsaicinoid contents varied among the examined cultivars (Table 1). Korean chili pepper (green) was found to contain 4.86 mg capsaicinoid/100 g fresh pepper, which increased to 9.71 mg in the ripe (red) peppers (Table 1). With approximately five times more capsaicinoids than Korean chili peppers (Table 1), Cheongyang chili peppers contained 27.87 mg/100 g of fresh peppers (253.35 mg/100 g of dried peppers). Shishito peppers had 1.96 mg capsaicinoid/100 g of fresh peppers. Similar to bell peppers, cucumber chili peppers exhibited an undetectable or negligible amount of capsaicinoids (Al Othman, Ahmed, Habila, & Ghafar, 2011).

**TABLE 1:** Capsaicinoid content of various chili pepper cultivars produced in Korea for fresh consumption

Cultivar	Dry weight basis (mg/100 g)			Moisture correction factor^a^	Wet weight basis (mg/100 g)			Reference
	Capsaicin	Dihydrocapsaicin	Capsaicinoid		Capsaicin	Dihydrocapsaicin	Capsaicinoid	
Korean chili pepper (“Nokguang gochu”), green	32.03	16.61	48.64	0.10	3.20	1.66	4.86	Jeon and Lee (2009)
Korean chili pepper, red	38.07	19.04^b^	57.11	0.17	6.47	3.24	9.71	Lee et al. (2011)
Chengyang chili pepper (“Cheongyang gochu”)	278.81	72.01	350.82	0.11	30.67	7.92	38.59	Jeon and Lee (2009)
	200	60^b^	260	0.11	22.00	6.60	28.60	Chung and Kang (1985)
	195.27^c^	67.01^c^	262.28^c^	0.11	21.48	7.37	28.85	Hwang et al. (2011)
	117.30	22.98	140.28	0.11	12.90	2.53	15.43	Park et al. (2019)
Mean	195.27	55.50	253.35		21.76	6.11	27.87	
Shishito pepper					0.66	0.48	1.14	Kim, Kim, Lee, and Lee (1996)
	15.35	7.79	23.14	0.12	1.84	0.93	2.78	Park et al. (2019)
Mean							1.96	
Cucumber chili pepper (Oi gochu)	0.00	0.00	0.00	0.08	0.00	0.00	0.00	Park et al. (2019)

^a^ The moisture correction factor was calculated using the following formula: (100 – fresh pepper water content)/ (100 – dried pepper water content).

^b^ Dihydrocapsaicin levels were estimated using the same ratio of capsaicin to dihydrocapsaicin for a given cultivar derived from other studies that analyzed both levels.

^c^ The value represents an average of capsaicinoid levels measured from peppers cultivated in 13 different regions.

For comparison, orange habanero peppers (*C. chinense*) contain approximately 180 and 1,280 mg of capsaicinoids per 100 g of fresh and dried peppers, respectively (Kurian & Starks, 2002). A similar level of 1,035 mg per 100 g was also reported for dried orange habaneros (Garces‐Claver, Arnedo‐Andres, Abadia, Gil‐Ortega, & Alvarez‐Fernandez, 2006). Tabasco pepper contains 625 mg per 100 g of dried peppers (Garces‐Claver et al., 2006). Therefore, Cheongyang chili pepper, the hottest chili pepper cultivar produced in Korea, contains lower capsaicinoid concentrations than Tabasco or habanero peppers.

### 3.2 Red pepper powder

Many cultivars of chili peppers are produced in Korea. Most chili peppers are cultivated for the production of red pepper powder, which is obtained after a drying process (Kim et al., 2002). Red pepper powder is traditionally made from sun‐dried Korean red chili peppers or ripened Cheongyang chili peppers. Additionally, many other chili pepper varieties are cultivated for drying and red pepper powder production. Capsaicinoid content can vary depending on the cultivar, area of cultivation, temperature, sun exposure, and storage conditions (Hwang et al., 2011; Kim et al., 2002; Lee et al., 2013; Si, Man, Chen, & Chung, 2014; Wang et al., 2009). Differences in these factors lead to variations in capsaicinoid levels in red pepper powder.

**FIGURE 2:**
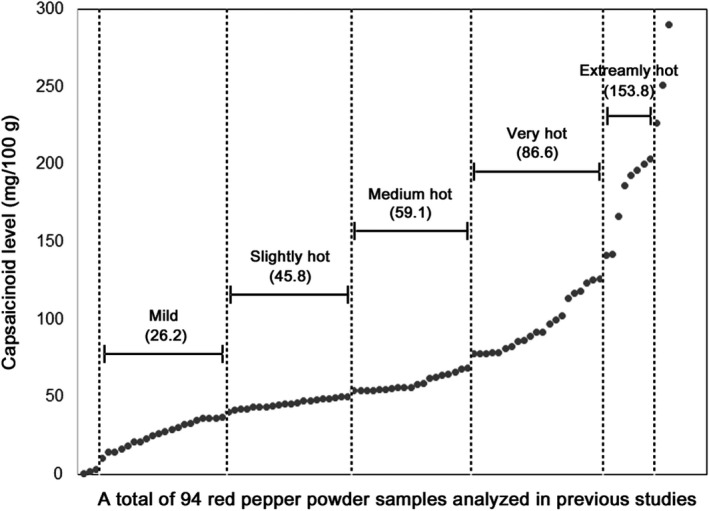
Capsaicinoid levels in red pepper powder, as determined by previous studies that analyzed various red pepper powders produced in Korea. Capsaicinoid levels were divided into five groups after the first and last three values were excluded due to their exceptionally low and high levels, respectively. The mean capsaicinoid value (mg/100 g red pepper powder) is shown for each of the five groups

After a comprehensive literature search to determine the capsaicinoid content in red pepper powders produced in Korea, this study identified a total of six studies that used GC or HPLC to analyze the capsaicinoid content of various red pepper powders consumed in Korea. One study provided only a mean capsaicinoid content (35.3 mg/100 g red pepper powder) for a variety of red pepper powders (Dang et al., 2018). Individual values of a total of 94 capsaicinoid contents (mg/100 g) were obtained from five additional studies (Choi, Jeon, & Park, 2000; Ham et al., 2012; Kim et al., 2002; Ku, Lee, & Park, 2012; Yu et al., 2009), which are listed in Table S1 and are shown in increasing order in Figure 2.

The first (<10 mg/100 g) and last (>220 mg/100 g) three values were excluded due to their exceptionally low or high levels. The resulting capsaicinoid levels (10.54–203.8 mg/100 g) were divided into five groups: 0.54–36.81 (mild, *n = *20; mean: 26.2 mg/100 g), 40.29–50.04 (slightly hot, *n = *20; mean: 45.8 mg/100 g), 53.99–68.8 (medium hot, *n = *19; mean: 59.1 mg/100 g), 77.7–102.3 (very hot, *n = *15; mean: 86.6 mg/100 g), and 113.52–203.8 mg/100 g (extremely hot, *n = *14; mean: 153.8 mg/100 g). Therefore, five levels containing a mean of 26.2, 45.8, 59.1, 86.6, and 153.8 mg capsaicinoid/100 g red pepper powder were determined for mild, slightly hot, medium hot, very hot, and extremely hot red pepper powders, respectively (Figure 2).

The capsaicinoid levels derived from two studies with relatively large sample sizes of 46 and 15 samples were found to be well‐distributed within the five aforementioned divisions (Kim et al., 2002; Ku et al., 2012). Capsaicinoid levels derived from another two studies were distributed in all categories, except for the mild category (Choi et al., 2000; Yu et al., 2009). In contrast, the red pepper powders analyzed by Ham et al. (*n* = 6) (Ham et al., 2012) fell within the mild and extremely hot categories only (Table S1). These results may have been due to a sample size limitation rather than analytical bias. Even after removing the six values derived from Ham et al. study, the overall distribution remained largely similar (data not shown). Dang et al. reported only a mean value of 35.5 mg capsaicinoid/100 g red pepper powder for 101 samples collected from different regions of Korea (Dang et al., 2018), which was found to be between the mild and slightly hot categories defined in this study.

The five capsaicinoid levels determined in this study (Figure 2) correlated well with the range established by another study, which analyzed capsaicinoid levels in four red pepper powders (sold in local markets) classified by spiciness. The capsaicinoid levels were found to be 20.8, 70.9, 106.7, and 145.3 mg/100 g for mild, medium, very hot, and extremely hot red pepper powders, respectively (Yu et al., 2009). Therefore, the capsaicinoid levels in red pepper powders determined from the literature in this study accurately reflected the capsaicinoid levels in a variety of red pepper powders consumed in Korea. These capsaicinoid concentrations in red pepper powders were used to estimate the capsaicinoid content in other food items that contained red pepper powder as their major capsaicinoid sources.

### 3.3 Hot sauce

KNHANES‐surveyed hot sauces included sweet hot chili sauce, Tabasco hot sauce, and chicken seasoning sauce. The capsaicinoid content in sweet hot chili sauce was estimated to be 1.6 mg/100 g sauce, averaged from the capsaicinoid contents of two products (Ham et al., 2012). For the Tabasco sauce, only one product is predominant in the Korean market, and it was reported to contain 20 mg capsaicinoid/100 g sauce (Betts, 1999). No reports were available for chicken seasoning sauce, and therefore, sweet hot chili sauce values were used instead due to their similar spiciness (Table 2).

**TABLE 2:** Capsaicinoid content in hot sauce

	Capsaicin (mg/100 g)	Dihydrocapsaicin (mg/100 g)	Capsaicinoid (mg/100 g)	Reference
Mcllhhenny's tabasco sauce	12	8	20	Betts (1999)
Chili sauce	1.44	0.90	2.34	Ham et al. (2012)
	0.49	0.37	0.86	
Mean^a^			1.60	

^a^ Estimation was also used for chicken seasoning sauce.

### 3.4 Kimchi

In Korea, kimchi is a popular side dish of salted and fermented vegetables. Kimchi types primarily vary by the main vegetables used for their preparation, and these include napa cabbage, Korean radish, young radish, cucumber, green onion, and many other vegetables. With the exception of white kimchi, kimchi is typically prepared with varying quantities of red pepper powder with varying degrees of spiciness. Some types of kimchi include water in their preparations, and these are properly referred to as “watery kimchi” (Kong et al., 2005). Watery kimchi contains less red pepper powder than typical kimchi (without added water) (Kong et al., 2005). A total of 11 types of kimchi were retrieved from the KNHANES 24‐hr recall datasets. These included napa cabbage kimchi, cubed Korean radish kimchi, young radish kimchi, watery kimchi, ponytail radish kimchi, mustard green kimchi, cucumber kimchi, green onion kimchi, rapeseed leaf kimchi, and sonchus‐leaf crepidiastrum kimchi.

Standardized recipes for different types of kimchi were searched, and standardized recipes for napa cabbage kimchi were found to be available (Cho, Park, & Rhee, 1997; Choi, Hwang, & Jo, 1997), in addition to cubed Korean radish kimchi (Choi et al., 1997), young radish kimchi (Kong et al., 2005), and watery kimchi (Kong et al., 2005), which are more commonly consumed than other types of kimchi. For these studies, red pepper powder proportions were calculated based on the main ingredient total weights (Table 3). Kimchi preparation typically includes salting and draining of the main vegetable ingredients (cabbage or radish). When standardized recipes only provided fresh cabbage or radish weights, these were corrected for weight loss (approximately 10%) due to moisture loss during the salting and draining processes (Han & Seok, 1998). Additionally, capsaicinoid content is reportedly to be decreased by microorganisms‐mediated degradation (Lee et al., 2015). For instance, capsaicinoid content decreased by approximately 20% (10%–25%) in napa cabbage kimchi during fermentation (Ku et al., 2004). Considering that red pepper powder is a major source of capsaicinoids in kimchi, the red pepper content was further corrected to reflect the level of capsaicinoid degradation during fermentation (Table 3).

**TABLE 3:** Estimation of red pepper powder content in various kimchi products

	Main vegetables (g)	Radish (g)	Green onion (g)	Red pepper powder (g)	Garlic (g)	Ginger (g)	Salted seafood/ Fish sauce (g)	Water (g)	Total (g)	Red pepper content (%)^a^	Corrected red pepper content (%)^b^	Reference
Napa cabbage	100	13.0	2.0	3.5	1.4	0.6	2.2	–	122.7	2.9		Cho et al. (1997)
	90^c^	11.4	2.1	1.5	0.9	0.4	3.0	–	109.3	1.4		Choi et al. (1997)
Mean										2.2	1.76	
Cubed Korean radish	90^c^		10.4	2.4	2.1	0.9	4.3	–	110.1	2.2	1.76^d^	Choi et al. (1997)
Watery kimchi	45	26.9	1.9	0.7	1.2	0.9	1.0	100	176.6	0.4	0.32	Kong et al. (2005)
Young radish kimchi	100		8.0	4.2	2.9	1.6	3.7	–	120.4	3.5	2.80^e^	Kong et al. (2005)

^a^ Red pepper powder content was calculated by dividing the total weight of the main ingredients by the weight of the red pepper powder.

^b^ Red pepper powder content was corrected for capsaicinoid loss during fermentation (approximately 20%).

^c^ Weight was corrected for water loss (10%) during salting and draining.

^d^ Estimation was also used for cucumber kimchi.

^e^ Estimation was also used for ponytail radish kimchi, mustard green kimchi, green onion kimchi, rapeseed leaf kimchi, and sonchus‐leaf crepidiastrum kimchi.

Standardized recipes were unavailable for other types of kimchi, and therefore, their red pepper powder content was estimated from that of other kimchi made from vegetables with similar mechanical properties. For example, the red pepper powder content of cubed Korean radish kimchi was used to estimate that of cucumber kimchi, and that of young radish kimchi was used to estimate red pepper powder content in the remaining types of kimchi (ponytail radish kimchi, mustard green kimchi, green onion kimchi, rapeseed leaf kimchi, and sonchus‐leaf crepidiastrum kimchi) (Table 3).

The National Agricultural Products Quality Management Service (NAQS) established a Good Food Post‐Certification System that offers certification for Korean traditional products, including kimchi, when they meet the standards and specifications for Korean traditional food. According to the NAQS, napa cabbage kimchi can either be claimed to be mild, moderately hot, or hot when its capsaicinoid content is <0.4, 0.4–1.2, and >1.2 mg/100 g, respectively. To determine whether estimations in this study fell within these ranges, the capsaicinoid content was estimated in kimchi using five levels of capsaicinoids in red pepper powder (Figure 2) and identified red pepper powder contents in kimchi products (Table 3), resulting in a capsaicinoid content range of 0.47, 0.81, 1.04, 1.52, or 2.71 mg/100 g kimchi, depending on the capsaicinoid contents in red pepper powder (Table 4). These results confirmed that the capsaicinoid contents determined herein were consistent with napa cabbage kimchi products consumed in Korea. Ku et al. reported a 0.2–1.35 mg capsaicinoid/100 g napa cabbage kimchi (Ku et al., 2004), which was also comparable to the levels of capsaicinoids estimated in this study. For comparison, another study reported 0.33 mg capsaicinoids/100 g napa cabbage kimchi, which was averaged from 121 samples (Dang et al., 2018). However, it is worth noting that the kimchi was first rinsed to remove the red pepper powder in that study, which could have led to an underestimation of its capsaicinoid content.

**TABLE 4:** Estimation of capsaicinoid content in various kimchi products based on the red pepper content in kimchi products (Table 3) and five levels of capsaicinoid contents in red pepper powder (Figure 2)

	Red pepper powder (%)	Capsaicinoid level (mg/100 g)				
		Mild	Slightly hot	Medium hot	Very hot	Extremely hot
Napa cabbage	1.76	0.47	0.81	1.04	1.52	2.71
Cubed Korean radish	1.76	0.47	0.81	1.04	1.52	2.71
Watery kimchi	0.32	0.09	0.15	0.19	0.28	0.49
Young radish kimchi	2.80	0.74	1.28	1.65	2.42	4.31

### 3.5 Salted seafood

Salted seafood (“jeotgal”) or fish sauce (liquid “jeot”) is salted and fermented dish made from seafood, such as shrimp, oyster, clam, squid, fish, or fish roe. Unlike other salted seafood products (e.g., salted shrimp and salted anchovy), some salted seafood products are seasoned with red pepper powder (e.g., salted oysters and salted squid). Among the various salted seafood items that contain red pepper powder, a total of seven were found to be consumed by Koreans, according to the 2012–2016 KNHANES. These included salted oysters, pollock roe, squid, baby squid, octopus, fish gills, and fish guts.

Presently, salted seafood is more often purchased than prepared at home. The capsaicinoid content from this food category was estimated using the red pepper powder content listed on the food label provided by each product's respective manufacturers (Table 5). Red pepper powder contents were available for salted oysters, pollock roe, squid, octopus, and fish guts (Table 5). With the exception of salted pollock roe (3.2%), salted oysters, squid, octopus, and fish guts were found to contain approximately 5% of red pepper powder (Table 5). Therefore, a 5% red pepper powder content was assumed to estimate the capsaicinoid content in the aforementioned food items, except the salted pollock roe. This 5% criterion was also used for the two remaining salted seafood items for which red pepper powder content information was unavailable (salted baby squid and salted fish gills) due to their similar preparation (Table 5). The capsaicinoid contents for red pepper powder were estimated at five levels of spiciness (Figure 2), resulting in 1.33, 2.29, 2.95, 4.33, and 7.69 mg/100 g salted seafood, except for salted pollock roe which it was estimated to contain either 0.84, 1.45, 1.87, 2.74, or 4.86 mg/100 g (Table 5).

**TABLE 5:** Estimation of red pepper powder and capsaicinoid content in salted seafood

Product number	1	2	3	4	5	6	7	Mean	Estimation	Capsaicinoid content (mg/100g)^a^				
										Mild	Slightly hot	Medium hot	Very hot	Extremely hot
Salted pollack roe	2.5^b^	2.0	2.5	2.5	4.3	4.0	4.3	3.2	3.2	0.84	1.45	1.87	2.74	4.86
Salted oyster	5.0	4.0	6.5	4.0	7.0	5.0		5.3	5.0^c^	1.33	2.29	2.95	4.33	7.69
Salted squid	6.0	5.4	4.0	4.0				4.8						
Salted octopus	6.0	5.0						5.5						
Salted fish gut	6.2	3.0	4.0	5.0				4.6						
Salted baby squid								5.0^c^						
Salted fish gill								5.0^c^						

^a^ Capsaicinoid contents were estimated based on the red pepper content in the salted seafood products and the capsaicinoid content in red pepper powder at five levels (Figure 2).

^b^ Red pepper powder content (%) was identified from food labels provided by the manufacturers.

^c^ A 5% red pepper powder content was assumed for all salted seafood except salted pollock roe.

### 3.6 Red pepper paste

Red pepper paste (“gochujang”) is a fermented condiment made from red pepper powder, glutinous rice, fermented soybean, barley malt powder, and salt. Although traditional red pepper paste is naturally fermented at home for several years, modern red pepper paste is produced commercially, and most Koreans purchase it at grocery stores or markets. Red pepper paste is extensively used in Korean cuisine and is also often mixed with other condiments such as vinegar or soy bean paste.

The red pepper powder content in commonly consumed products ranged between 9.3% and 12.5%, with 11.3% being the most typical proportion. Therefore, the red pepper powder content in red pepper paste was estimated at 11.3%. Additionally, changes in capsaicinoid content during fermentation were also accounted for. Similar to kimchi (Section 3.4), the capsaicinoid content of red pepper paste has been shown to decrease after preparation (Lee et al., 2015). This decrease in capsaicinoid amount continued throughout the 90‐day fermentation period, which typically results in a capsaicinoid loss of approximately 10% (Yang, Lee, & Choi, 2018). Therefore, the estimated red pepper powder content was reduced from 11.3% to 10.2% to reflect the decrease in capsaicinoids during fermentation (Table 6). When the five levels of capsaicinoid content in red pepper powder (Figure 2) were considered, the red pepper paste was estimated to contain either 2.71, 4.66, 6.00, 8.81, or 15.64 mg capsaicinoid/100 g if it was prepared with mild, slightly hot, medium hot, very hot, or extremely hot red pepper powder, respectively. This capsaicinoid content estimation in red pepper paste was comparable to the level of capsaicinoids measured in commercially available red pepper paste products. One study reported an average capsaicinoid content of 6.66 mg for 120 products commonly consumed in Korea (Dang et al., 2018) and another study reported a 3.92–5.90 mg capsaicinoids/100 g range in eight commercially available red pepper paste samples (Ham et al., 2012).

**TABLE 6:** Estimation of red pepper powder and capsaicinoid content in red pepper paste

	Red pepper powder (%)^a^	Corrected red pepper powder (%)^b^	Capsaicinoid content^c^ (mg/100 g)				
			Mild	Slightly hot	Medium hot	Very hot	Extremely hot
Red pepper paste (100%)	11.3	10.2	2.71	4.66	6.00	8.81	15.64
Red pepper paste (80%) with vinegar	9.0	8.14	2.16	3.73	4.80	7.05	12.51
Red pepper paste (25%) with soybean paste	2.8	2.54	0.68	1.16	1.50	2.20	3.91

^a^ Red pepper powder contents were identified from food labels.

^b^ Red pepper powder contents were corrected for capsaicinoid loss (10%) during fermentation.

^c^ Capsaicinoid contents were estimated based on the red pepper powder content in red pepper paste and the capsaicinoid content of red pepper powder at five levels (Figure 2).

The KNHANES separated gather information on the consumption of traditional and commercial red pepper paste. According to the KNHANES datasets, both types were found to be consumed. The capsaicinoid content in the traditional red pepper paste was 2.46–9.64 mg capsaicinoid/100 g red pepper paste among 12 samples, which was similar to that of commercial red pepper paste (Jo et al., 2013). The capsaicinoid content in red pepper paste in this study was thus assumed to be the same for both traditional and commercial products. This study also estimated the red pepper powder content and capsaicinoid content of red pepper paste containing vinegar and soybean paste by adjusting the red pepper paste proportions down to 80% and 25% in red pepper paste products with vinegar and soybean paste, respectively (Table 6).

### 3.7 Instant noodles

Instant noodles (“ramyeon”) are a popular food in Korea, particularly among young people (Kim et al., 2013; Yu, Jung, & Yoon, 2013). A variety of hot flavors are available in the market, along with new varieties that claim to be “extremely hot,” which highlights the current trend toward hot flavors. Hot flavors can originate from different combinations of chili peppers; however, this information is often proprietary. Instant noodles are often either packaged in a bag (regular noodles) or a cup (cup noodles). Typically, seasonings are packaged separately in a small packet and are provided in powder form for noodles served in soup or liquid form (sauce) for noodles not served in soup.

In this study, the capsaicinoid content of 24 seasonings (16 from regular noodles and 8 from cup noodles) was quantified using HPLC (Table 7). Seasoning size (weight) varied among the products, and therefore, the capsaicinoid content in a seasoning packet (one serving) was used to compare capsaicinoid contents in different instant noodles. The capsaicinoid contents in regular instant noodles could be categorized into three different flavor intensities: mild (*n = *6; mean: 0.70 mg/serving), hot (*n = *7; mean: 1.51 mg/serving), and extremely hot (*n = *3; mean: 5.60 mg/serving). Cup noodles tended to contain comparable or slightly higher capsaicinoid concentrations compared with the same noodles packaged in a bag (Table 7). The average capsaicinoid contents determined for three different hot flavor categories can be used to estimate the capsaicinoids in other instant noodles with similar spiciness.

**TABLE 7:** Capsaicinoid levels analyzed in instant noodle seasonings via HPLC

Instant noodle type	Flavor	Seasoning type	Manufacturer/ Brand	Capsaicin (mg/100 g)	Dihydrocapsaicin (mg/100 g)	Capsaicinoid (mg/100 g)	Weight of seasoning (g)	Capsaicinoid in a packet (mg/100 g)
Regular	Mild	Powder	NS NGR mild	3.80 ± 0.44^a^	1.57 ± 0.38	5.37 ± 0.7	10.6	0.57 ± 0.07
		Powder	SY SGK	2.79 ± 0.66	2.36 ± 0.09	5.15 ± 0.57	12.0	0.62 ± 0.07
		Powder	OTG Jin mild	4.11 ± 0.33	1.81 ± 0.12	5.92 ± 0.34	11.1	0.66 ± 0.04
		Liquid	OTG BBM	2.04 ± 0.09	0.51 ± 0.54	2.55 ± 0.45	28.8	0.73 ± 0.13
		Powder	SY SY	3.32 ± 0.45	3.43 ± 0.29	6.75 ± 0.17	12.2	0.82 ± 0.02
		Powder	NS AS	4.27 ± 0.24	2.20 ± 0.06	6.47 ± 0.19	12.2	0.79 ± 0.02
	Mean			3.39	1.98	5.37		0.70
	Hot	Powder	SY MPM	5.23 ± 0.85	2.89 ± 0.32	8.12 ± 0.76	12.3	1.00 ± 0.09
		Powder	SY MSN	5.20 ± 0.45	3.63 ± 0.05	8.83 ± 0.46	12.8	1.13 ± 0.06
		Liquid	PD BBM	3.26 ± 0.33	0.78 ± 0.56	4.04 ± 0.88	28.4	1.15 ± 0.25
		Powder	NS NGR hot	10.26 ± 0.74	5.54 ± 0.15	15.80 ± 0.62	10.5	1.66 ± 0.06
		Powder	OTG Jin hot	8.77 ± 0.48	6.27 ± 0.88	15.04 ± 0.92	11.4	1.71 ± 0.11
		Powder	NS OJE	9.25 ± 0.45	5.91 ± 1.71	15.16 ± 0.64	11.8	1.79 ± 0.08
		Powder	NS Shin	11.62 ± 1.24	8.40 ± 2.33	20.02 ± 1.11	10.5	2.10 ± 0.12
	Mean			7.66	4.77	12.43		1.51
	Extremely hot	Powder	OTG Yeol	15.40 ± 1.13	12.91 ± 2.39	28.31 ± 1.68	11.7	3.31 ± 0.20
		Powder	PD TS	26.65 ± 2.60	25.95 ± 3.64	52.60 ± 1.07	12.0	6.31 ± 0.13
		Liquid	SY BD	17.23 ± 0.95	8.31 ± 1.64	25.54 ± 0.71	28.1	7.18 ± 0.20
	Mean			19.76	15.72	35.48		5.60
Cup	Mild	Powder	NS YGJ	5.83 ± 0.33	3.33 ± 0.25	9.16 ± 0.56	8.2	0.75 ± 0.05
	Hot	Powder	SY MPM	6.46 ± 0.13	3.15 ± 0.08	9.61 ± 0.06	13.0	1.25 ± 0.01
		Powder	NS SW	6.63 ± 0.34	4.25 ± 0.54	10.88 ± 0.87	13.0	1.41 ± 0.11
		Powder	PD WTG	9.70 ± 0.84	4.93 ± 0.65	14.63 ± 1.36	11.0	1.61 ± 0.15
		Powder	NS OJE	7.39 ± 0.32	5.03 ± 0.34	12.42 ± 0.3	11.8	1.47 ± 0.05
		Powder	NS Shin	11.64 ± 1.01	5.10 ± 1.08	16.74 ± 2.00	14.0	2.34 ± 0.28
		Liquid	OTG RMB	4.85 ± 0.49	2.70 ± 0.57	7.55 ± 0.29	34.9	2.63 ± 0.10
		Liquid	OTG Jin hot	12.68 ± 1.49	7.78 ± 0.24	20.46 ± 1.73	13.1	2.68 ± 0.23
	Mean			8.48	4.71	13.18		1.91

^a^ All samples were analyzed in triplicate, and the results were expressed as mean ± *SD*.

### 3.8 Convenience foods other than instant noodles

Convenience foods other than instant noodles were also retrieved from the KNHANES dataset. These items included dumplings (kimchi dumpling), spicy canned tuna (hot pepper canned tuna), seasonings for stew‐like dishes, and spicy snacks. Capsaicinoids in stew‐like dish seasonings were mainly derived from red pepper powder and red pepper paste. However, various kinds of seasonings are commercially available, and the concentration of the seasoning depends on the amount of water used in its preparation. These situations made it challenging to estimate the amount of red pepper powder or other ingredients, and therefore, estimations could not be generalized for this food category. For spicy snack products, estimations were further complicated by the wide variety of products, in addition to their relatively short product life cycles. For this reason, seasonings for stew‐like dishes and spicy snacks were excluded from the CAPKO database.

Kimchi dumplings are frozen food products that contain kimchi. The kimchi content was identified from the respective food labels to determine the capsaicinoid content. The average kimchi content from eight widely consumed products was 23.52%. The average red pepper content in kimchi dumplings was calculated as 0.41% (Table 8) according to the red pepper content estimated for kimchi (Table 3). The resulting capsaicinoid content was 0.11, 0.19, 0.24, 0.36, or 0.64 mg/100 g when it was assumed to be prepared with mild, slightly hot, medium hot, very hot, and extremely hot red pepper powders, respectively (Figure 2).

**TABLE 8:** Estimation of capsaicinoid contents in kimchi dumplings

Manufacturer	Kimchi content (%)^a^	Red pepper powder content (%)^b^	Capsaicinoid content (mg/100 g)^c^				
			Mild	Slightly hot	Medium hot	Very hot	Extremely hot
CJ	9.83	0.17	0.046	0.080	0.102	0.150	0.266
	21.14	0.37	0.099	0.170	0.220	0.322	0.572
DW	22.31	0.39	0.104	0.180	0.232	0.340	0.603
	32.18	0.57	0.151	0.259	0.334	0.490	0.871
HT	25.0	0.44	0.117	0.202	0.260	0.381	0.677
	13.9	0.24	0.065	0.112	0.144	0.212	0.376
PMW	39.53	0.70	0.185	0.319	0.410	0.602	1.070
	24.23	0.43	0.113	0.195	0.252	0.369	0.656
Mean	23.52	0.41	0.110	0.190	0.244	0.358	0.637

^a^ Kimchi content was identified from the food labels provided by the manufacturers.

^b^ Red pepper content was derived from the kimchi content in Table 3.

^c^ Capsaicinoid content was estimated based on the red pepper powder content in kimchi dumplings and the capsaicinoid content in red pepper powder at five levels (Figure 2).

Spicy canned tuna contains red pepper powder and chili sauce as major capsaicinoid‐containing ingredients. Spicy canned tuna products contained 4.7%–6.2% red pepper powder and 16.3%–28% chili sauce (Table 9). The product with the higher red pepper content (OTG) had less chili sauce than the other products (Table 8). The capsaicinoid content determined for chili sauce (Table 2) and red pepper powder (Figure 2) was used to estimate the capsaicinoid contents in spicy canned tuna. The mean capsaicinoid content was estimated as 1.78, 2.80, 3.50, 4.96, or 8.52 mg/100 g of canned tuna, respectively, assuming 1.6% capsaicinoid content in chili sauce (Table 2) and five levels of capsaicinoids in red pepper powder (Figure 2). Capsaicinoids are known to undergo thermal degradation, especially at temperatures above 180°C (Si et al., 2014; Wang et al., 2009). However, heating temperatures between 100 and 190°C for 15 min did not substantially affect capsaicinoid contents (<10% reduction) (Wang et al., 2009). This suggests that canning, which is commonly achieved by maintaining the food core temperature at 121°C for a short period (Dincer, 1998), does not result in significant capsaicinoid degradation.

**TABLE 9:** Estimation of capsaicinoid content of spicy canned tuna

Manufacturer	Red pepper powder content (%)^a^	Chili sauce (%)^a^	Capsaicinoid content (mg/100 g)^b^				
			Mild	Slightly hot	Medium hot	Very hot	Extremely hot
DW	4.7	25.5	1.66	2.56	3.18	4.48	7.64
OTG	6.2	16.3	1.91	3.10	3.92	5.63	9.80
SZ	5.0	28.0	1.78	2.74	3.40	4.78	8.14
Mean			1.78	2.80	3.50	4.96	8.52

^a^ Information was obtained from the food label provided by the manufacturers.

^b^ Capsaicinoid contents were estimated by combining the capsaicinoid level derived from red pepper powder at five levels (Figure 2) and the capsaicinoid content in chili sauce (Table 2).

In this study, capsaicinoid‐containing foods commonly consumed in Korea were identified from the KNHANES datasets. Capsaicinoid levels of primary capsaicinoid sources were estimated from the literature, after which the capsaicinoid content of food categories that contained primary capsaicinoid sources could be estimated using primary capsaicinoid source contents identified from standard recipes or food labels. For the food categories for which the content of capsaicinoid sources could not be identified, capsaicinoid levels were analyzed via HPLC. Therefore, this study established a methodological framework for the development of databases for other compounds with potential health effects.

This study developed a database of capsaicinoid content in foods commonly consumed in Korea (CAPKO). Red pepper powder is a major source of capsaicinoids in the Korean diet. Individuals who favor spicy foods may consume high amounts of capsaicinoids by consuming products that contain red pepper powder, particularly if it has a high capsaicinoid content. The capsaicinoid content was determined at five levels (26.6, 45.8, 59.0, 86.6, and 153.8 mg/100 g) to reflect the diversity of concentrations found in red pepper powder. These five capsaicinoid levels were used to estimate the capsaicinoid content in foods that contain red pepper powder, including kimchi, salted seafood, red pepper paste, and convenience foods other than instant noodles. The capsaicinoid contents in napa cabbage kimchi and red pepper paste estimated in this study were validated by comparing them with previous capsaicinoid analysis data. The capsaicinoid contents in instant noodle seasonings were analyzed and categorized into three different spiciness intensities to obtain a more granular capsaicinoid consumption range. A limitation of the database developed in this study was that it does not cover all possible food items that contain capsaicinoids, particularly convenience foods. However, the KNHANES datasets were used to thoroughly identify capsaicinoid‐containing foods commonly consumed by Koreans. This comprehensive CAPKO database will allow for reliable estimations of capsaicinoid consumption in Korea to identify subpopulations or individuals who consume high capsaicinoid levels.

## CONFLICT OF INTEREST

The authors declare no conflicts of interest.

## ETHICAL STATEMENTS

This study did not involve any human or animal testing.

## Supporting information

Table S1
