# Supplementary material for: Development of a database of capsaicinoid contents in foods commonly consumed in Korea
Source: Food Sci Nutr. 2020 Jul 16;8(8):4611–24. doi: 10.1002/fsn3.1785 (PMC7455983; doi:10.1002/fsn3.1785)
Supplement: Supplementary file 1 — Table S1 [file FSN3-8-4611-s001.docx]

Supplementary Table S1. List of individual capsaicinoid values derived from previous studies that analyzed capsaicinoid contents in red pepper powder

|  | Capsaicin | Dihydrocapsaicin | Capsaicinoid | Reference |  |  | Capsaicin | Dihydrocapsaicin | Capsaicinoid | Reference |
| --- | --- | --- | --- | --- | --- | --- | --- | --- | --- | --- |
| 1 | 28.8 | 12.9 | 41.7 | (Choi, Jeon, & Park, 2000) |  | 48 | 5.69 | 4.85 | 10.54 | (Kim, Park, & Hwang, 2002) |
| 2 | 25.1 | 19.2 | 44.3 |  |  | 49 | 9 | 7.66 | 16.66 |  |
| 3 | 28.4 | 16.7 | 45.1 |  |  | 50 | 10.71 | 7.75 | 18.46 |  |
| 4 | 30.3 | 17.4 | 47.7 |  |  | 51 | 11.59 | 9.29 | 20.88 |  |
| 5 | 28.9 | 19.3 | 48.2 |  |  | 52 | 13.74 | 11.3 | 25.04 |  |
| 6 | 32.6 | 23.6 | 56.2 |  |  | 53 | 15.11 | 12.59 | 27.7 |  |
| 7 | 34.5 | 23.8 | 58.3 |  |  | 54 | 16.49 | 12.48 | 28.97 |  |
| 8 | 38.3 | 24 | 62.3 |  |  | 55 | 16.27 | 14.06 | 30.33 |  |
| 9 | 38.4 | 30.4 | 68.8 |  |  | 56 | 18.64 | 14.07 | 32.71 |  |
| 10 | 46 | 31.7 | 77.7 |  |  | 57 | 19.76 | 15.17 | 34.93 |  |
| 11 | 47.7 | 30.6 | 78.3 |  |  | 58 | 22.05 | 13.91 | 35.96 |  |
| 12 | 50.3 | 35.9 | 86.2 |  |  | 59 | 21.2 | 15.29 | 36.49 |  |
| 13 | 86.2 | 39.9 | 126.1 |  |  | 60 | 21.21 | 15.29 | 36.5 |  |
| 14 | 1.8 | 1.18 | 2.98 | (Ham et al., 2012) |  | 61 | 20.34 | 19.95 | 40.29 |  |
| 15 | 9.17 | 5.44 | 14.61 |  |  | 62 | 38.97 | 3.4 | 42.37 |  |
| 16 | 12.38 | 8.49 | 20.87 |  |  | 63 | 24.03 | 18.47 | 42.5 |  |
| 17 | 15.47 | 7.66 | 23.13 |  |  | 64 | 24.72 | 19.09 | 43.81 |  |
| 18 | 18.7 | 13.58 | 32.28 |  |  | 65 | 22.28 | 23.82 | 46.1 |  |
| 19 | 78.24 | 38.82 | 117.06 |  |  | 66 | 24.99 | 22.62 | 47.61 |  |
| 20 | 0.54 | 0 | 0.54 | (Ku, Lee, & Park, 2012) |  | 67 | 26.13 | 22.51 | 48.64 |  |
| 21 | 1.84 | 0 | 1.84 |  |  | 68 | 28.46 | 20.4 | 48.86 |  |
| 22 | 7.52 | 6.84 | 14.36 |  |  | 69 | 30.49 | 19.2 | 49.69 |  |
| 23 | 13.02 | 13.06 | 26.08 |  |  | 70 | 26.99 | 22.94 | 49.93 |  |
| 24 | 19.11 | 17.7 | 36.81 |  |  | 71 | 25.86 | 24.18 | 50.04 |  |
| 25 | 35.88 | 9.7 | 45.58 |  |  | 72 | 28.14 | 25.85 | 53.99 |  |
| 26 | 31.39 | 22.91 | 54.3 |  |  | 73 | 25.62 | 28.63 | 54.25 |  |
| 27 | 41.75 | 26.21 | 67.96 |  |  | 74 | 34.15 | 20.8 | 54.95 |  |
| 28 | 51.52 | 26.27 | 77.79 |  |  | 75 | 32.58 | 22.89 | 55.47 |  |
| 29 | 59.76 | 29.66 | 89.42 |  |  | 76 | 34.44 | 21.68 | 56.12 |  |
| 30 | 69.69 | 32.61 | 102.3 |  |  | 77 | 34.56 | 24.16 | 58.72 |  |
| 31 | 76.42 | 49.41 | 125.83 |  |  | 78 | 38.81 | 24.08 | 62.89 |  |
| 32 | 91.46 | 50.93 | 142.39 |  |  | 79 | 38.97 | 26.9 | 65.87 |  |
| 33 | 124.3 | 68.82 | 193.12 |  |  | 80 | 43.17 | 34.67 | 77.84 |  |
| 34 | 185.01 | 105.15 | 290.16 |  |  | 81 | 45.51 | 33.28 | 78.79 |  |
| 35 | 31.63 | 11.96 | 43.59 | (Yu, Choi, & Lee, 2009) |  | 82 | 44.09 | 37.04 | 81.13 |  |
| 36 | 28.23 | 15.48 | 43.71 |  |  | 83 | 45.55 | 40.89 | 86.44 |  |
| 37 | 25.18 | 20.42 | 45.6 |  |  | 84 | 51.34 | 40.74 | 92.08 |  |
| 38 | 38.92 | 15.19 | 54.11 |  |  | 85 | 52.52 | 39.63 | 92.15 |  |
| 39 | 29.82 | 24.84 | 54.66 |  |  | 86 | 56.57 | 40.26 | 96.83 |  |
| 40 | 34.42 | 21.44 | 55.86 |  |  | 87 | 65.55 | 47.97 | 113.52 |  |
| 41 | 43.98 | 20.06 | 64.04 |  |  | 88 | 68.16 | 55.4 | 123.56 |  |
| 42 | 45.19 | 19.46 | 64.65 |  |  | 89 | 101.99 | 64.69 | 166.68 |  |
| 43 | 55.36 | 27.39 | 82.75 |  |  | 90 | 122.95 | 73.05 | 196 |  |
| 44 | 70.62 | 29.05 | 99.67 |  |  | 91 | 115.49 | 84.5 | 199.99 |  |
| 45 | 84 | 33.94 | 117.94 |  |  | 92 | 127.63 | 76.17 | 203.8 |  |
| 46 | 95.97 | 45.12 | 141.09 |  |  | 93 | 119.83 | 106.63 | 226.46 |  |
| 47 | 123.62 | 62.88 | 186.5 |  |  | 94 | 129.93 | 120.93 | 250.86 |  |
